# Molnupiravir or nirmatrelvir–ritonavir plus usual care versus usual care alone in patients admitted to hospital with COVID-19 (RECOVERY): a randomised, controlled, open-label, platform trial

**DOI:** 10.1016/S1473-3099(25)00093-3

**Published:** 2025-05-15

**Authors:** RECOVERY Collaborative Group, Peter W Horby, Natalie Staplin, Leon Peto, Jonathan R Emberson, Mark Campbell, Guilherme Pessoa-Amorim, Buddha Basnyat, Louise Thwaites, Rogier van Doorn, Raph L Hamers, Jeremy Nel, John Amuasi, Richard Stewart, Dipansu Ghosh, Natalie Blencowe, Purav Desai, Nicholas Easom, Jaydip Majumdar, Paul Hine, David Chadwick, Graham Cooke, Rahuldeb Sarkar, Hanif Esmail, J Kenneth Baillie, Maya H Buch, Saul N Faust, Thomas Jaki, Edmund Juszczak, Katie Jeffery, Marian Knight, Wei Shen Lim, Alan Montgomery, Aparna Mukherjee, Andrew Mumford, Kathryn Rowan, Guy Thwaites, Marion Mafham, Richard Haynes, Martin J Landray

## Abstract

**Background:**

Molnupiravir and nirmatrelvir–ritonavir are oral antivirals that have shown efficacy in preventing disease progression in outpatients with COVID-19. We aimed to evaluate these treatments for patients hospitalised with COVID-19 pneumonia, for whom data on these antivirals are scarce.

**Methods:**

The RECOVERY trial is a randomised, controlled, open-label, adaptive platform trial testing treatments for COVID-19. In this study we report the molnupiravir and nirmatrelvir–ritonavir comparisons from the RECOVERY trial. In each comparison, participants aged 18 years and older were randomly allocated (1:1) to the relevant antiviral (5 days of molnupiravir 800 mg twice daily or 300 mg nirmatrelvir and 100 mg ritonavir twice daily) in addition to usual care, or to usual care alone. The molnupiravir comparison was conducted at 75 hospitals in the UK, two in Nepal, and two in Indonesia; the nirmatrelvir–ritonavir comparison was conducted at 32 hospitals in the UK. Participants could take part in both comparisons. The primary outcome was 28-day mortality, and secondary outcomes were time to discharge alive from hospital and progression to invasive ventilation or death. Analysis was by intention to treat. Both comparisons were stopped because of low recruitment. This study is registered with ISRCTN, 50189673, and ClinicalTrials.gov, NCT04381936.

**Findings:**

From Jan 24, 2022, to May 24, 2023, 923 participants were recruited to the molnupiravir comparison (445 allocated to molnupiravir and 478 to usual care), and from March 31, 2022, to May 24, 2023, 137 participants were recruited to the nirmatrelvir–ritonavir comparison (68 allocated to nirmatrelvir–ritonavir and 69 to usual care). More than three-quarters of participants were vaccinated and had antispike antibodies at randomisation, and more than two-thirds were receiving other SARS-CoV-2 antivirals. In the molnupiravir comparison, 74 (17%) participants allocated to molnupiravir and 79 (17%) allocated to usual care died within 28 days (hazard ratio [HR] 0·93 [95% CI 0·68–1·28], p=0·66). In the nirmatrelvir–ritonavir comparison, 13 (19%) participants allocated to nirmatrelvir–ritonavir and 13 (19%) allocated to usual care died within 28 days (HR 1·02 [0·47–2·23], p=0·96). In neither comparison was there evidence of any difference in the duration of hospitalisation or the proportion of participants progressing to invasive ventilation or death.

**Interpretation:**

Adding molnupiravir or nirmatrelvir–ritonavir to usual care was not associated with improvements in clinical outcomes. However, low recruitment meant a clinically meaningful benefit of treatment could not be ruled out, particularly for nirmatrelvir–ritonavir.

**Funding:**

UK Research and Innovation (UK Medical Research Council), the National Institute for Health and Care Research, and the Wellcome Trust.

## Introduction

Early antiviral treatment of unvaccinated individuals at high risk of severe COVID-19 can substantially reduce the risk of subsequent hospitalisation or death.^1–3^ There is less evidence supporting antiviral treatment in people admitted to hospital, and in these patients it might be that immune-mediated lung damage, rather than ongoing viral replication, is primarily responsible for disease progression. Antiviral treatment with neutralising monoclonal antibodies has been shown to substantially reduce mortality in hospitalised patients, but only in those not yet producing their own anti-SARS-CoV-2 antibodies.^4^ However, most immunocompetent adults now have some SARS-CoV-2 immunity following vaccination or previous infection, and available neutralising monoclonal antibodies are now largely ineffective because of spike-gene mutations in globally prevalent SARS-CoV-2 variants.^5,6^ In previous studies,^7,8^ remdesivir, a nucleoside analogue inhibitor of the viral RNA-dependent RNA polymerase, reduced time to discharge by around 1 day in hospitalised patients and was associated with a moderate reduction in mortality in non-ventilated patients. Other SARS-CoV-2 antivirals, including molnupiravir (Lagevrio; MSD, Rahway, NJ, USA) and nirmatrelvir–ritonavir (Paxlovid; Pfizer, New York, NY, USA), have not been adequately tested in randomised trials in hospitalised patients, and it could be that these drugs, given alone or in combination with other antivirals, improve clinical outcomes.

Molnupiravir is an orally absorbed prodrug of N(4)-hydroxycytidine, a nucleoside-analogue substrate of the viral RNA-dependent RNA polymerase. Molnupiravir has a broad spectrum of activity against RNA viruses, including coronaviruses, and a high barrier to the development of viral resistance.^9–11^ The drug’s mechanism of action is distinct to remdesivir, impairing viral RNA replication by facilitating ambiguous base pairing, leading to an accumulation of transition mutations (so-called error catastrophe). This accumulation impairs viral replication and increases the rate of viral clearance, but there are concerns that molnupiravir-induced viral mutagenesis could encourage the emergence of new variants with increased transmissibility or reduced susceptibility to antivirals.^12^ In the MOVe-OUT trial, early treatment of unvaccinated individuals with COVID-19 at high risk of disease progression reduced the risk of hospitalisation or death by 30% (risk ratio [RR] 0·70 [95% CI 0·49–0·99], p=0·045), but no significant benefit was shown in the subsequent PANORAMIC trial among vaccinated individuals infected with omicron variants at lower risk of disease progression (RR 1·07 [0·81–1·41], p=0·62).^13,14^ MOVe-IN, which included 304 unvaccinated individuals from 15 countries between October, 2020, and January, 2021, is the only reported trial of molnupiravir in hospitalised patients.^15^ This trial found no difference in the primary outcome of recovery by day 29 (82–85% in the molnupiravir groups *vs* 85% in the placebo group) or mortality (6% in the molnupiravir group *vs* 3% in the placebo group) but was underpowered to rule out a clinically meaningful improvement in either outcome.

Nirmatrelvir is an orally administered small-molecule inhibitor of the viral 3-chymotrypsin-like protease, which is co-administered with ritonavir to enhance its pharmacokinetics.^16^ In the EPIC-HR trial of unvaccinated individuals with early COVID-19 at high risk of progression to severe disease, nirmatrelvir–ritonavir reduced the risk of hospitalisation or death by 88% (RR 0·12 [95% CI 0·06–0·25], p<0·0001), although no significant benefit was identified in the subsequent EPIC-SR trial of individuals who were vaccinated and at lower risk (RR 0·48 [0·17–1·41], p=0·18).^3,17^ Only one trial has reported nirmatrelvir–ritonavir use in hospitalised patients, which included 264 individuals, who were largely unvaccinated, recruited in China in April–May, 2022.^18^ In that trial there was no difference in the primary outcome of 28-day mortality (4% in the nirmatrelvir–ritonavir group *vs* 6% in the standard treatment group), but the trial was underpowered to rule out a clinically meaningful benefit of treatment.

In this study we aimed to carry out independent evaluations of molnupiravir and nirmatrelvir–ritonavir plus usual care versus usual care alone in patients hospitalised with COVID-19 pneumonia during the omicron era.

## Methods

### Study design and participants

The Randomised Evaluation of COVID-19 Therapy (RECOVERY) trial is an investigator-initiated, individually randomised, controlled, open-label, adaptive platform trial to evaluate the effects of potential treatments in patients hospitalised with COVID-19. Details of the trial design and results for other treatments have been published previously.^4,19–27^ The trial was conducted at hospital organisations in the UK—supported by the National Institute for Health and Care Research Clinical Research Network—and in south and southeast Asia and Africa. Of these, 75 hospitals in the UK, two in Nepal, and two in Indonesia enrolled participants in the molnupiravir comparison, and 32 UK hospitals enrolled participants in the nirmatrelvir–ritonavir comparison (appendix pp 2–31). The trial was coordinated by the Nuffield Department of Population Health at the University of Oxford (Oxford, UK), the trial sponsor. The trial was conducted in accordance with the principles of the International Conference on Harmonisation Good Clinical Practice guidelines and was approved by all relevant regulatory authorities and ethics committees in each participating country (appendix p 32). The protocol, statistical analysis plan, and additional information are available online.

For the **study protocol, statistical analysis plan, and additional information** see https://www.recoverytrial.net/

Patients admitted to hospital were eligible for the study if they had SARS-CoV-2 infection confirmed by a PCR or antigen test, a pneumonia syndrome thought to be related to COVID-19, and no medical history that might, in the opinion of the managing physician, put the patient at significant risk if they were to participate in the trial. Patients were excluded from the molnupiravir comparison if they were pregnant or breastfeeding, or if they had received molnupiravir during their current illness. Patients were excluded from the nirmatrelvir–ritonavir comparison if they were in the first trimester of pregnancy (in the absence of extensive experience of use of the drug in pregnancy, women in the second or third trimesters were felt to be the group among whom the potential benefits most clearly outweighed any theoretical risks), had severe liver impairment (Child–Pugh class C), had severe renal impairment (estimated glomerular filtration rate <30 mL/min per 1·73 m^2^), had received nirmatrelvir–ritonavir during their current illness, or were receiving a concomitant medication with CYP3A4-dependent metabolism that risked a severe drug–drug interaction with nirmatrelvir–ritonavir. Children (aged <18 years) and those unable to take medication orally were excluded from both comparisons. If a study treatment was unavailable, or if the managing physician considered a study treatment to be either definitely indicated or definitely contraindicated, participants were excluded from the relevant comparison. Written informed consent was obtained from all participants, or from a legal representative if participants were too unwell or otherwise unable to provide informed consent.

The trial is registered with ISRCTN, 50189673, and ClinicalTrials.gov, NCT04381936.

### Randomisation and masking

Participants could enter either one or both of the comparisons provided they were eligible. For each comparison they entered, participants were randomly assigned (1:1) to either usual standard of care plus the relevant treatment or usual standard of care without the relevant treatment, using web-based simple (unstratified) randomisation with allocation concealed until after randomisation (appendix pp 41–43). Participants allocated to molnupiravir were to receive 800 mg orally twice daily for 5 days. Participants allocated to nirmatrelvir–ritonavir were to receive 300 mg nirmatrelvir and 100 mg ritonavir orally twice daily for 5 days, reduced to 150 mg and 100 mg twice daily if they had moderate renal impairment (estimated glomerular filtration rate 30–59 mL/min per 1·73 m^2^). In both comparisons, the course was to be continued after discharge if it was incomplete.

As a platform trial, and in a factorial design, participants could be simultaneously randomised to other concurrently evaluated treatment groups: empagliflozin versus usual care, higher-dose corticosteroids versus usual care, or sotrovimab versus usual care (appendix pp 41–42). Participants and local study staff were not masked to allocated treatment. Other than members of the data monitoring committee, all individuals involved in the trial were masked to aggregated outcome data while recruitment and 28-day follow-up were ongoing.

### Procedures

Baseline data were collected using a web-based case report form that included demographics, level of respiratory support, major comorbidities, suitability of the study treatment for a particular participant, COVID-19 vaccination status, and study treatment availability at the study site (appendix pp 43–46). A serum sample and nose swab were collected at randomisation from UK participants and sent to central laboratories for testing. Serum was tested for anti-SARS-CoV-2 spike antibodies, anti-SARS-CoV-2 nucleocapsid antibodies, and SARS-CoV-2 nucleocapsid antigen using Roche Elecsys assays (Roche Diagnostics, Basel, Switzerland). Participants were classified as positive or negative for antispike and antinucleocapsid antibodies using manufacturer-defined thresholds, and as positive or negative for serum nucleocapsid antigen using the study population median value (as this assay had not previously been validated on serum samples). Nose swabs were tested for SARS-CoV-2 RNA using TaqPath COVID-19 RT-PCR (Thermo Fisher Scientific, Waltham, MA, USA). Samples with sufficient concentration of viral RNA were sequenced using the ONT Midnight protocol (Oxford Nanopore Technologies, Oxford, UK).^28^ Sequence data were used to detect mutations associated with resistance to molnupiravir or nirmatrelvir–ritonavir identified from literature searches. Further details of laboratory analyses are in the appendix (pp 32–33).

Follow-up nose swabs were collected from UK participants on days 3 and 5 (counting the day of randomisation as day 1). These swabs were analysed in the same manner as the baseline swab described above.

A single online follow-up form was completed when participants were discharged or had died, or at 28 days after randomisation, whichever occurred first (appendix pp 47–55). Information was recorded on adherence to allocated study treatment, receipt of other COVID-19 treatments, duration of admission, receipt of respiratory or renal support, and vital status (including cause of death). In addition, in the UK, routine healthcare and registry data were obtained, including information on vital status (with date and cause of death), discharge from hospital, receipt of respiratory support, renal replacement therapy, and participant ethnicity. For sites outside the UK, a further case report form (appendix pp 56–57) collected vital status at day 28 (if not already reported on the initial follow-up form).

Molnupiravir and nirmatrelvir–ritonavir were supplied by the UK Government in the UK and bought from commercial suppliers in Nepal and Indonesia.

### Outcomes

Outcomes were assessed at 28 days after randomisation, with further analyses specified at 6 months. The primary outcome was all-cause mortality at 28 days. Secondary outcomes were time to discharge from hospital and, among participants not on invasive mechanical ventilation at randomisation, invasive mechanical ventilation (including extracorporeal membrane oxygenation) or death. Prespecified subsidiary clinical outcomes were use of non-invasive respiratory support, time to successful cessation of invasive mechanical ventilation (defined as cessation of invasive mechanical ventilation within, and survival to, 28 days), use of renal dialysis or haemofiltration, cause-specific mortality, bleeding events, thrombotic events, major cardiac arrhythmias, non-SARS-CoV-2 infections, and metabolic complications (including ketoacidosis). Prespecified virological outcomes were viral RNA copy number in nose swabs taken at days 3 and 5, and the frequency of detection of resistance markers. Information on suspected serious adverse reactions was collected in an expedited manner to comply with regulatory requirements.

### Statistical analysis

Because trial recruitment and event rates during the COVID-19 pandemic were unpredictable, RECOVERY treatment comparisons do not have a predetermined sample size. With high levels of recruitment, the intention would have been to continue until enough primary outcomes had accrued to have 90% power to detect a proportional risk reduction of 20% with a two-sided p value of 0·01 (approximately 5500 participants if mortality was 20% without treatment).

Following the initial wave of omicron infection in the UK in early 2022, the number of patients hospitalised with COVID-19 pneumonia reduced substantially in the UK, as did recruitment to both comparisons. Because of persistently low recruitment, the RECOVERY trial steering committee decided to close both comparisons on May 24, 2023, while still masked to the results. Based on the final recruitment and event rates described below, the molnupiravir comparison had 29% power, and the nirmatrelvir–ritonavir comparison had 9% power, to detect a 20% proportional reduction in 28-day mortality with a two-sided p value of 0·05 or less.

The primary analysis for all outcomes was by intention to treat, comparing participants randomly assigned to the study treatment with participants randomly assigned to usual care but for whom the study treatment was both available and suitable as a treatment. For the primary outcome of 28-day mortality, the hazard ratio (HR) from an age-adjusted and respiratory-status-adjusted Cox model was used to estimate the mortality HR. Kaplan–Meier survival curves were constructed to display cumulative mortality over the 28-day period. The same Cox regression method was used to analyse time to hospital discharge and successful cessation of invasive mechanical ventilation, with patients who died in hospital right-censored on day 29. Median time to discharge was derived from Kaplan–Meier estimates. For the prespecified composite secondary outcome of progression to invasive mechanical ventilation or death within 28 days (among those not receiving invasive mechanical ventilation at randomisation), and the subsidiary clinical outcomes of receipt of invasive or non-invasive ventilation, or use of haemodialysis or haemofiltration, the precise dates were not available and so a log-binomial regression model was used to estimate the risk ratio (RR) adjusted for age and respiratory status. SARS-CoV-2 viral RNA copy number in nose swabs were estimated with ANCOVA using the log-transformed values after adjustment for each participant’s baseline value, age, and level of respiratory support at randomisation.

Prespecified subgroup analyses were performed for the primary outcome using the statistical test of interaction (test for heterogeneity or trend), in accordance with the prespecified analysis plan, defined by the following characteristics at randomisation: age, sex, ethnicity, level of respiratory support, days since symptom onset, and use of corticosteroids (appendix p 135). Exploratory subgroup analyses were also performed by SARS-CoV-2 antibody status (anti-S and anti-N), serum nucleocapsid antigen status, and use of other antivirals (appendix pp 154–55).

Estimates of HRs and RRs are shown with 95% CIs, calculated assuming normality of parameter estimates. All p values are two-sided and are shown without adjustment for multiple testing. The full database is held by the study team, which collected the data from study sites and performed the analyses at the Nuffield Department of Population Health, University of Oxford. Analyses were performed using SAS version 9.4 and R version 3.4.

### Role of the funding source

Neither the funders nor the manufacturers of molnupiravir or nirmatrelvir–ritonavir had any role in study design, data collection, data analysis, data interpretation, or writing of the report.

## Results

**Figure 1:**
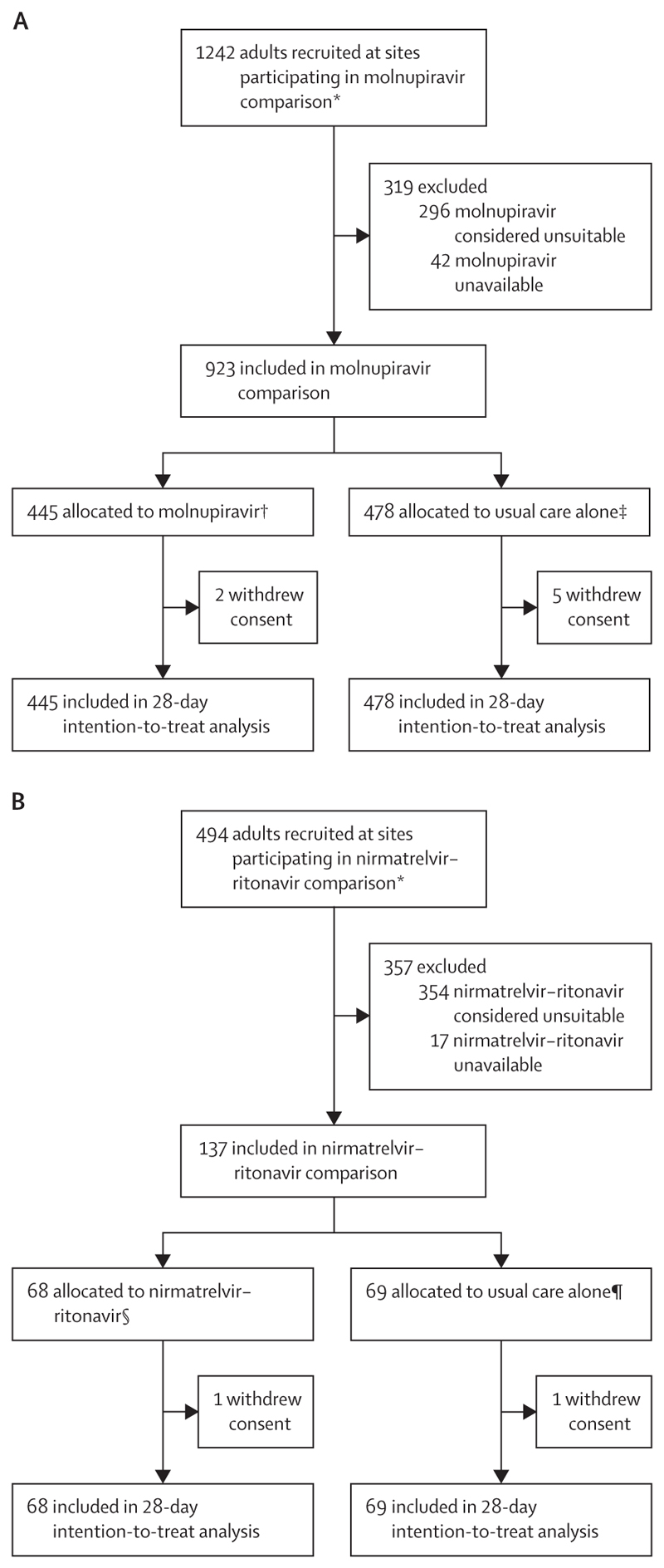
Trial profile Molnupiravir comparison (A) and nirmatrelvir–ritonavir comparison (B). Drug unavailability and unsuitability are not mutually exclusive. *During the period in which participants could be recruited to the comparison. †413 of 443 patients with completed follow-up forms at the time of analysis received molnupiravir. ‡None of 472 patients with completed follow-up forms at time of analysis received molnupiravir. §60 of 67 patients with completed follow-up forms at time of analysis received nirmatrelvir−ritonavir. ¶None of 68 patients with completed follow-up forms at time of analysis received nirmatrelvir−ritonavir.

Baseline characteristics of participants in this study are shown in table 1. Between Jan 24, 2022, and May 24, 2023, 923 (74%) of 1242 patients enrolled in RECOVERY at sites participating in the molnupiravir comparison were eligible to be randomly allocated to molnupiravir, of whom 445 were allocated to molnupiravir and 478 were allocated to usual care without molnupiravir (figure 1A). The 319 RECOVERY participants not included in the molnupiravir comparison had similar characteristics to those included (appendix p 60). The mean age of study participants in this comparison was 71·4 years (SD 14·1), 767 (83%) had received a COVID-19 vaccine, and the median time since symptom onset was 5 days (IQR 3–9). 133 (14%) of 923 participants in the molnupiravir comparison simultaneously participated in the nirmatrelvir–ritonavir comparison. At randomisation, 809 (88%) participants were receiving corticosteroids and 629 (68%) were receiving, or allocated to receive, a SARS-CoV-2 antiviral other than molnupiravir (including remdesivir as part of usual care and sotrovimab or nirmatrelvir–ritonavir allocated in RECOVERY). 227 (25%) participants were anti-N seropositive and 705 (76%) were anti-S seropositive (table 1).

**Figure 2:**
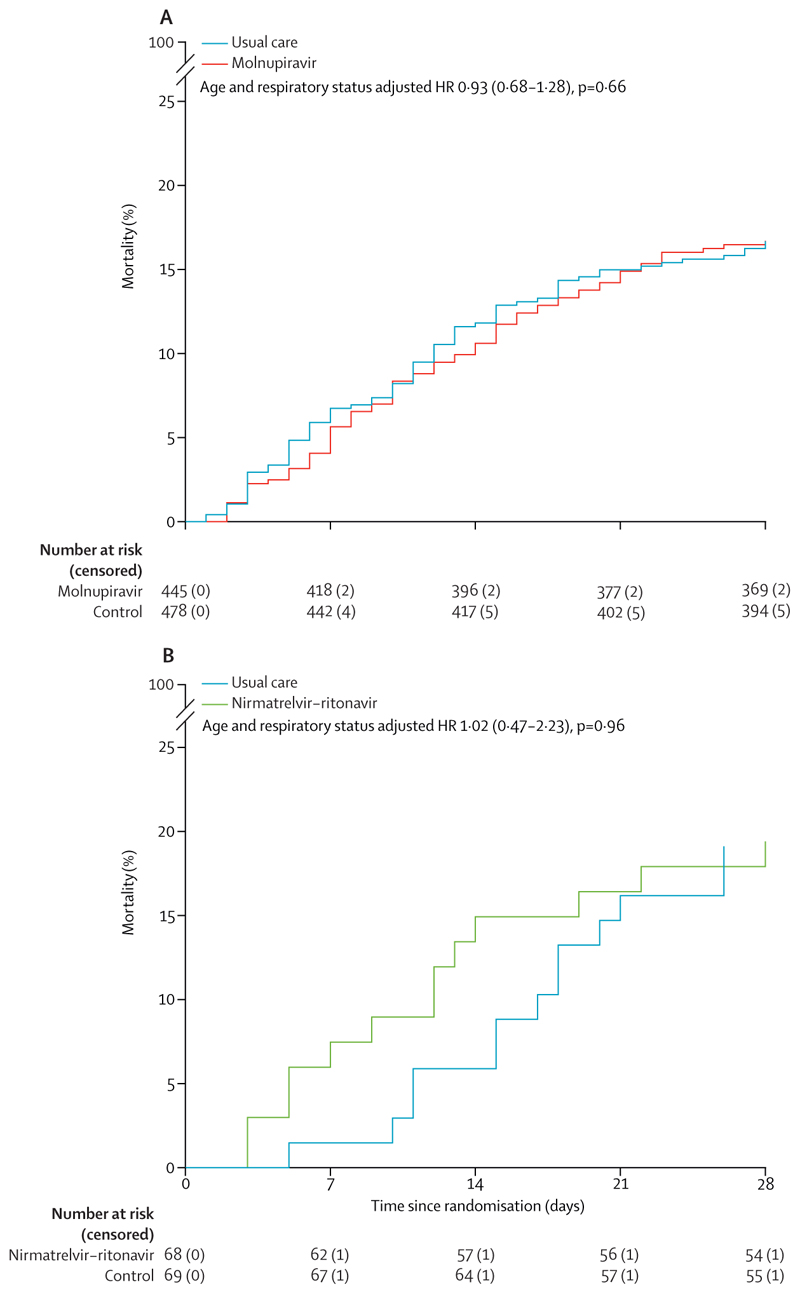
Cumulative mortality over 28 days in the molnupiravir (A) and nirmatrelvir–ritonavir (B) comparisons HR=hazard ratio (95% CI).

**Figure 3:**
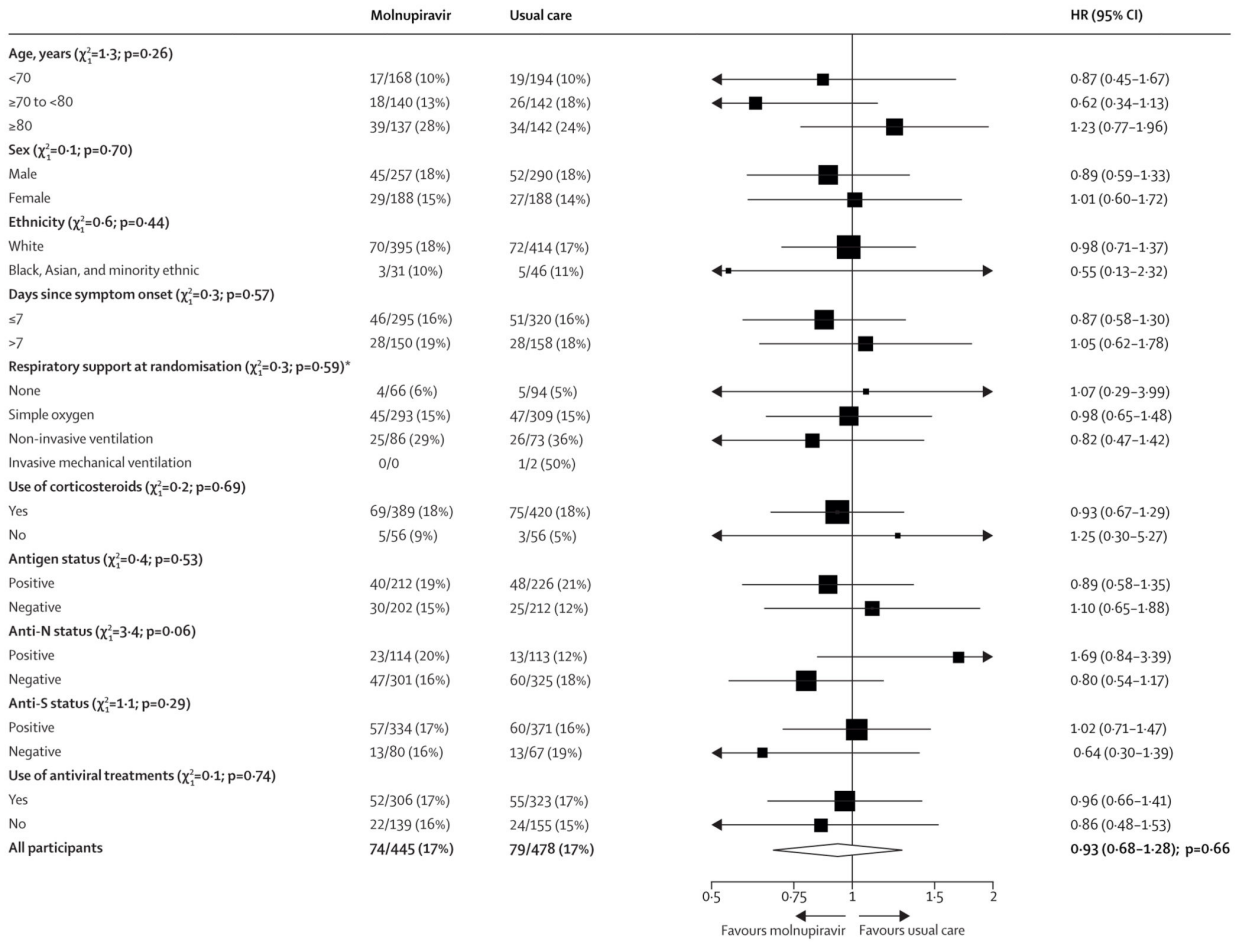
Subgroup analyses for the primary outcome (28-day mortality) in the molnupiravir comparison Subgroup-specific HRs are represented by squares (with areas proportional to the amount of statistical information) and the lines through them correspond to 95% CIs. The subgroups of ethnicity, days since symptom onset, and use of corticosteroids exclude those with missing data. HR=hazard ratio. *Trend test does not include invasive mechanical ventilation due to insufficient numbers of patients.

The follow-up form was completed for 915 (99%) participants in the molnupiravir comparison; among them, 413 (93%) of 443 in the molnupiravir group received at least one dose of molnupiravir, compared with none of 472 in the usual care group (appendix p 64). Primary and secondary outcome data are known for more than 99% of randomly assigned participants. There was no evidence of a significant difference in the proportion of participants who met the primary outcome of 28-day mortality between the two randomised groups (74 [17%] in the molnupiravir group *vs* 79 [17%] in the usual care group; HR 0·93 [95% CI 0·68–1·28], p=0·66; table 2, figure 2A). We observed similar results in all prespecified subgroups, and in exploratory subgroups defined by serum SARS-CoV-2 antigen or antibody status and use of other SARS-CoV-2 antiviral treatments (figure 3).

There was no evidence of a significant difference in the time to discharge alive within 28 days in the molnupiravir comparison (319 [72%] *vs* 354 [74%] discharged; HR 0·96 [95% CI 0·82–1·12], p=0·60; table 2). Among those not on invasive mechanical ventilation at baseline, the number of participants progressing to the prespecified composite secondary outcome of invasive mechanical ventilation or death was similar in both groups (77 [17%] of 445 *vs* 81 [17%] of 476; RR 0·96 [0·73–1·25], p=0·75). Similar results were seen in all prespecified subgroups of participants (appendix pp 68–69).

We found no evidence of differences in prespecified subsidiary clinical outcomes or cause-specific mortality between groups (table 2, appendix p 65). There were more episodes of hyperglycaemia requiring insulin in participants allocated to molnupiravir versus usual care (33 [7%] of 445 *vs* 15 [3%] of 478; absolute difference 4% [95% CI 1–7], p=0·0038; appendix p 66). The rates of other safety outcomes were similar between groups, including new cardiac arrhythmia, thrombotic events, clinically significant bleeds, non-coronavirus infections, seizures, acute liver injury, and acute kidney injury (appendix p 66). There were no reported suspected serious adverse reactions in participants allocated to molnupiravir.

872 (98%) of 893 UK participants had at least one nose swab available for analysis. Compared with usual care, allocation to molnupiravir was associated with a lower viral RNA copy number in nose swabs taken on day 5 (–0·45 log_10_ copies per mL [95% CI –0·74 to –0·16], p=0·0024), but not on day 3 (table 2). 622 (67%) participants had at least one successfully sequenced sample with 90% or higher genome coverage, and of these, 620 (>99%) were omicron variants (primarily BA.1, BA.2, BA.5, and XBB). No candidate molnupiravir resistance mutations were identified from literature searches; therefore, it was not possible to evaluate baseline or follow-up nose swabs for mutations associated with resistance.

Between March 31, 2022, and May 24, 2023, 137 (28%) of 494 participants recruited at sites participating in the nirmatrelvir–ritonavir comparison were eligible to be randomly allocated to nirmatrelvir–ritonavir, of whom 68 were allocated to nirmatrelvir–ritonavir and 69 to usual care without nirmatrelvir–ritonavir (figure 1B). The 357 RECOVERY participants not included in the nirmatrelvir–ritonavir comparison had similar characteristics to those included (appendix p 62). The mean age of study participants in this comparison was 72·5 years (SD 13·9), 116 (85%) had received a COVID-19 vaccine, and the median time since symptom onset was 4 days (IQR 3–8). 133 (97%) participants in the nirmatrelvir–ritonavir comparison also participated in the molnupiravir comparison. At randomisation, 122 (89%) participants were receiving corticosteroids, and 111 (81%) were receiving, or allocated to receive, a SARS-CoV-2 antiviral other than nirmatrelvir–ritonavir (including remdesivir as part of usual care, and sotrovimab or molnupiravir allocated in RECOVERY). 40 (29%) participants were anti-N seropositive and 112 (82%) were anti-S seropositive.

The follow-up form was completed for 135 (99%) participants in the nirmatrelvir–ritonavir comparison; among them, 60 (90%) of 67 in the nirmatrelvir–ritonavir group received at least one dose of nirmatrelvir–ritonavir, compared with none of 68 in the usual care group (appendix p 64). Primary and secondary outcome data are known for more than 99% of randomly assigned participants. There was no evidence of a significant difference in the proportion of participants who met the primary outcome of 28-day mortality between the two randomised groups (13 [19%] participants in the nirmatrelvir–ritonavir group *vs* 13 [19%] participants in the usual care group; HR 1·02 [95% CI 0·47–2·23], p=0·96; table 2, figure 2B). Because of low recruitment to this comparison, no subgroup analyses were performed.

There was no evidence of a significant difference in the time to discharge alive within 28 days in the nirmatrelvir–ritonavir comparison (48 [71%] *vs* 54 [78%] discharged; HR 0·80 [95% CI 0·54–1·20], p=0·29; table 2). Among those not on invasive mechanical ventilation at baseline, the number of participants progressing to the prespecified composite secondary outcome of invasive mechanical ventilation or death was similar in both groups (14 [21%] of 68 *vs* 13 [19%] of 69; RR 1·06 [0·54–2·08], p=0·86).

We found no evidence of differences in prespecified subsidiary clinical outcomes or cause-specific mortality between groups (table 2, appendix p 65). The rates of all safety outcomes were similar between groups (appendix p 66). There were no reported suspected serious adverse reactions in participants allocated to nirmatrelvir–ritonavir.

All participants had at least one nose swab available for analysis. Allocation to nirmatrelvir–ritonavir was associated with a lower viral RNA copy number in nose swabs taken on day 5 (–0·76 log_10_ copies per mL [95% CI –1·41 to –0·12], p=0·022), but not on day 3 (table 2). 97 (71%) participants had at least one sample successfully sequenced with 90% or higher genome coverage, and of these, 96 (99%) were omicron variants. No sequenced samples contained mutations at the 20 nucleotide positions in the 3-chymotrypsin-like protease that had previously been associated with a more than 2·5-fold median reduction in inhibition by nirmatrelvir.

## Discussion

In these two reported evaluations from the RECOVERY trial, among patients admitted to hospital for severe COVID-19, neither molnupiravir nor nirmatrelvir–ritonavir were found to reduce mortality, duration of hospitalisation, or the risk of being ventilated or dying for those not on ventilation at baseline. However, both comparisons had insufficient statistical power to exclude modest differences in these outcomes, particularly for the primary outcome of mortality. There was more certainty in estimates of the secondary outcome of time to discharge, with 95% CIs excluding an HR greater than 1·12 for molnupiravir and greater than 1·20 for nirmatrelvir–ritonavir. For context, previous effective treatments evaluated in RECOVERY have included tocilizumab, with time-to-discharge HR 1·22 (95% CI 1·12–1·33), and casirivimab–imdevimab, with time-to-discharge HR 1·19 (1·09–1·31) among seronegative patients.^4,23^

Previous trials have indicated the potential benefit of antiviral treatment with neutralising monoclonal antibodies or remdesivir in hospitalised patients, but randomised evidence has been inadequate for molnupiravir and nirmatrelvir–ritonavir, two widely available antivirals with efficacy shown in early SARS-CoV-2 infection.^3,4,8,14^ For each drug, only one other randomised trial in hospitalised patients has been reported to date, but neither was large enough to detect plausibly moderate benefits of treatment.^15,18^ The present RECOVERY comparisons were both stopped because of low recruitment before they had reached the planned sample size, with 923 participants recruited to the molnupiravir comparison and 137 recruited to the nirmatrelvir–ritonavir comparison. Our results do not suggest any benefit in adding these antivirals to routine care, but the restricted recruitment means we cannot exclude a benefit.

The incidence of COVID-19 pneumonia has reduced substantially following widespread vaccination starting in 2021 and the global dominance of omicron SARS-CoV-2 variants in 2022. In this context, infection with SARS-CoV-2 in hospitalised patients is often an incidental finding or is associated with non-respiratory illness, and the benefits of antiviral therapy in this setting might be modest. By contrast, RECOVERY only included participants with pneumonia thought to be related to COVID-19. In 770 (83%) of the 927 participants, this had developed despite previous COVID-19 vaccination; in keeping with this, only around one-quarter of participants were antispike antibody negative at baseline, but around three-quarters were anti-nucleocapsid antibody negative, indicating that this was their first SARS-CoV-2 infection.

The power to perform subgroup analyses was restricted even in the molnupiravir comparison, for which there was no strong signal of a differential effect of treatment in participants by antibody status, level of serum viral antigen, use of other antiviral treatments, symptom duration, or severity of illness. In participants allocated to molnupiravir, there was an excess of hyperglycaemia requiring insulin compared with usual care, reported in 33 versus 15 participants. An excess of hyperglycaemia was also reported in the MOVe-IN trial (nine events *vs* one event), but there is no apparent mechanism to explain this observation, and it might represent a chance finding. The increased viral clearance in day 5 nose swabs seen in those allocated to molnupiravir is in keeping with its known antiviral activity and with results from trials in early infection, but this was not shown to translate into clinical benefit in RECOVERY.^13,15,29,30^

Recruitment to the nirmatrelvir–ritonavir comparison was substantially lower than the molnupiravir comparison, reflecting its introduction just after the initial wave of omicron infection in the UK in early 2022, the involvement of fewer hospital sites, and a high proportion of people for whom it was considered unsuitable. Reasons for unsuitability were not systematically recorded, but this was frequently related to potential interactions between ritonavir and concomitant medications. We were, therefore, unable to reliably assess whether nirmatrelvir–ritonavir improves clinical outcomes, although a reduction in viral RNA copy number among participants allocated to nirmatrelvir–ritonavir was observed.

The strengths of this trial are that it was randomised, it had broad eligibility criteria, there was baseline characterisation of markers of SARS-CoV-2 immune status and infection, and more than 99% of participants were followed up for the primary outcome. However, the restricted sample size does not allow us to exclude clinically meaningful benefits of the treatments tested. Additionally, use of other antiviral treatments was common in both comparisons, and it is possible that the treatments tested would have had a greater effect in the absence of other antivirals. As such, this trial principally evaluated the benefit of routinely adding these antivirals to usual care in which other antivirals were available. Although this randomised trial is open label (ie, participants and local hospital staff were aware of the assigned treatment), the primary and secondary outcomes are unambiguous and were ascertained without bias through linkage to routine health records in the large majority of participants. However, detailed information on radiological or physiological outcomes was not collected. No adjustment was made for multiple testing when calculating p values for subsidiary outcomes, which should be taken into account when interpreting day 5 viral RNA copy number results. 88% of participants were White, and the large majority were recruited in the UK, so the trial population does not mirror the global population of patients admitted to hospital with COVID-19. The RECOVERY trial only studied patients who had been hospitalised with COVID-19 and, therefore, is not able to provide any evidence on the safety and efficacy of these antivirals used in other patients with less severe infection. Due to the recommendation that both drugs be taken orally, and not via a gastric feeding tube, few participants were recruited who required invasive mechanical ventilation.

In summary, among adults hospitalised with COVID-19, most of whom were receiving antiviral therapy, the addition of molnupiravir or nirmatrelvir–ritonavir to usual care was not associated with reductions in 28-day mortality, duration of hospital stay, or progression to invasive ventilation or death. However, low recruitment means a clinically meaningful benefit of treatment cannot be ruled out, particularly for nirmatrelvir–ritonavir.

## Supplementary Material

Supplementary appendix

## Figures and Tables

**Table 1:** Baseline characteristics of participants

	Molnupiravir *vs* usual care		Nirmatrelvir-ritonavir *vs* usual care	
	Molnupiravir (n=445)	Usual care (n=478)	Nirmatrelvir-ritonavir (n=68)	Usual care (n=69)
Age, years	71·2 (14·3)	71·6 (14·0)	75·8 (13·1)	69·3 (14·1)
<70	168 (38%)	194 (41%)	18 (26%)	30 (43%)
70–79	140 (31%)	142 (30%)	26 (38%)	22 (32%)
≥80	137 (31%)	142 (30%)	24 (35%)	17 (25%)
Sex				
Male	257 (58%)	290 (61%)	41 (60%)	36 (52%)
Female*	188 (42%)	188 (39%)	27 (40%)	33 (48%)
Country				
Indonesia	4 (1%)	8 (2%)	0	0
Nepal	7 (2%)	11 (2%)	0	0
UK	434 (98%)	459 (96%)	68 (100%)	69 (100%)
Ethnicity				
White	395 (89%)	414 (87%)	61 (90%)	60 (87%)
Black, Asian, and minority	31 (7%)	46 (10%)	3 (4%)	4 (6%)
ethnic				
Unknown	19 (4%)	18 (4%)	4 (6%)	5 (7%)
Number of days since	5 (3–9)	5 (3–10)	4 (3–9)	5 (3–8)
symptom onset				
Number of days since	2 (1–4)	2 (1–4)	2 (1–4)	2 (1–5)
hospitalisation				
Respiratory support received				
None	66 (15%)	94 (20%)	13 (19%)	8 (12%)
Simple oxygen	293 (66%)	309 (65%)	40 (59%)	52 (75%)
Non-invasive ventilation	86 (19%)	73 (15%)	15 (22%)	9 (13%)
Invasive mechanical	0	2 (<1%)	0	0
ventilation				
Biochemistry				
C-reactive protein, mg/L	65 (25–135)	73 (33–137)	93 (41–148)	76 (33–167)
Creatinine, μmol/L	81 (64–112)	79 (64–110)	76 (64–104)	72 (59–97)
Previous diseases				
Diabetes	122 (27%)	126 (26%)	13 (19%)	20 (29%)
Heart disease	146 (33%)	163 (34%)	11 (16%)	17 (25%)
Chronic lung disease	183 (41%)	197 (41%)	22 (32%)	31 (45%)
Tuberculosis	1 (<1%)	1 (<1%)	0	1 (1%)
HIV	4 (1%)	2 (<1%)	0	1 (1%)
Severe liver disease^†^	11 (2%)	6 (1%)	0	0
Severe kidney impairment^‡^	33 (7%)	44 (9%)	0	0
Severely	96 (22%)	87 (18%)	14 (21%)	15 (22%)
immunocompromised^§^				
Any of the above	335 (75%)	375 (78%)	42 (62%)	55 (80%)
Received a COVID-19 vaccine	376 (84%)	391 (82%)	57 (84%)	59 (86%)
Use of other treatments				
Corticosteroids	389 (87%)	420 (88%)	59 (87%)	63 (91%)
Remdesivir	178 (40%)	194 (41%)	27 (40%)	31 (45%)
Tocilizumab	49 (11%)	56 (12%)	12 (18%)	13 (19%)
Plan to use tocilizumab	40 (9%)	32 (7%)	2 (3%)	8 (12%)
within the next 24 h				
Randomly assigned treatments in RECOVERY				
High-dose steroids	69 (16%)	87 (18%)	0	0
Empagliflozin	143 (32%)	138 (29%)	19 (28%)	20 (29%)
Sotrovimab	199 (45%)	221 (46%)	30 (44%)	35 (51%)
Molnupiravir	445 (100%)	0	34 (50%)	32 (46%)
Nirmatrelvir-ritonavir	34 (8%)	33 (7%)	68 (100%)	0
Viral RNA copy number in baseline nose swab				
Median level (log viral copies/mL)	6 (4–7)	6 (4–7)	6 (4–7)	6 (4–7)
Antigen status				
Positive	212 (48%)	226 (47%)	38 (56%)	35 (51%)
Negative	202 (45%)	212 (44%)	27 (40%)	34 (49%)
Unknown	31 (7%)	40 (8%)	3 (4%)	0
Serostatus (anti-N)				
Positive	114 (26%)	113 (24%)	21 (31%)	19 (28%)
Negative	301 (68%)	325 (68%)	44 (65%)	50 (72%)
Unknown	30 (7%)	40 (8%)	3 (4%)	0
Serostatus (anti-S)				
Positive	334 (75%)	371 (78%)	53 (78%)	59 (86%)
Negative	80 (18%)	67 (14%)	12 (18%)	10 (14%)
Unknown	31 (7%)	40 (8%)	3 (4%)	0

Data are mean (SD), n (%), or median (IQR).

* Includes no pregnant individuals.

† Defined as requiring ongoing specialist care.

‡ Defined as estimated glomerular filtration rate <30 mL/min per 1·73 m^2^.

§ In the opinion of the managing clinician.

**Table 2:** Key study outcomes

	Molnupiravir *vs* usual care				Nirmatrelvir-ritonavir *vs* usual care			
	Molnupiravir (n=445)	Usual care (n=478)	HR, RR, or MD (95% CI)	p value	Nirmatrelvir-ritonavir (n=68)	Usual care (n=69)	HR, RR, or MD (95% CI)	p value
28-day mortality (primary outcome)	74 (17%)	79 (17%)	HR 0·93 (0·68 to 1·28)	0·66	13 (19%)	13 (19%)	HR 1·02 (0·47 to 2·23)	0·96
Median time to discharge alive, days (secondary outcome)	10 (6 to >28)	9 (5 to >28)	··	··	10 (6 to >28)	8 (5 to 21)	··	
Discharged from hospital within 28 days (secondary outcome)	319 (72%)	354 (74%)	HR 0·96 (0·82 to 1·12)	0·60	48 (71%)	54 (78%)	HR 0·80 (0·54 to 1·20)	0·29
Receipt of invasive mechanical ventilation or death (secondary outcome)*	77/445 (17%)	81/476 (17%)	RR 0·96 (0.73 to 1.25)	0·75	14/68 (21%)	13/69 (19%)	RR 1·06 (0·54 to 2·08)	0·86
Invasive mechanical ventilation	8/445 (2%)	6/476 (1%)	RR 1·27 (0·45 to 3·60)	0·65	1/68 (1%)	1/69 (1%)	··	··
Death	74/445 (17%)	78/476 (16%)	RR 0·96 (0·73 to 1·26)	0·77	13/68 (19%)	13/69 (19%)	RR 0·98 (0·49 to 1·94)	0·94
Receipt of ventilation†	38/359 (11%)	34/403 (8%)	RR 1·24 (0·80 to 1·92)	0·34	4/53 (8%)	10/60 (17%)	RR 0·56 (0·18 to 1·73)	0·31
Non-invasive ventilation	35/359 (10%)	34/403 (8%)	RR 1·14 (0·73 to 1·78)	0·58	4/53 (8%)	10/60 (17%)	RR 0·56 (0·18 to 1·73)	0·31
Invasive mechanical ventilation	5/359 (1%)	1/403 (<1%)	RR 5·66 (0·66 to 48·37)	0·11	0/53 (<1%)	1/60 (2%)	··	··
Successful cessation of invasive mechanical ventilation‡	0/0	0/2	··	··	0/0	0/0	··	··
Renal replacement therapy§	5/436 (1%)	9/469 (2%)	RR 0·62 (0·20 to 1·86)	0·39	0/68	1/69 (1%)	··	··
Mean baseline-adjusted viral RNA copy number on day 3 (log copies/mL)	4·37 (0·08)	4·48 (0·09)	MD –0.11 (–0·36 to 0·13)	0·37	4·01 (0·23)	4·45 (0·23)	MD –0·44 (–1·07 to 0·19)	0·18
Mean baseline-adjusted viral RNA copy number on day 5 (log copies/mL)	3·57 (0·11)	4·02 (0·10)	MD –0·45 (–0·74 to -0·16)	0·0024	2·88 (0·24)	3·64 (0·22)	MD –0·76 (–1·41 to –0·12)	0·022

Data are n (%), n/N (%), median (IQR), or mean (SD), unless otherwise indicated. Viral RNA copy number measurements on days 3 and 5 were available for 653 (73%) and 574 (64%) of 893 UK participants in the molnupiravir comparison, and for 106 (77%) and 107 (78%) of 137 UK participants in the nirmatrelvir–ritonavir comparison. HR=hazard ratio. MD=mean difference. RR=risk ratio.

* Analyses exclude those on invasive mechanical ventilation at randomisation.

† Analyses exclude those on any form of ventilation at randomisation.

‡ Analyses restricted to those on invasive mechanical ventilation at randomisation.

§ Analyses exclude those on haemodialysis or haemofiltration at randomisation.

## Data Availability

The protocol, consent form, statistical analysis plan, information on definitions and derivation of clinical characteristics and outcomes, training materials, regulatory documents, and other relevant study materials are available online. As described in the protocol, the trial steering committee will facilitate the use of the study data and approval will not be unreasonably withheld. De-identified participant data will be made available to researchers registered with an appropriate institution within 3 months of publication. However, the steering committee will need to be satisfied that any proposed publication is of high quality, honours the commitments made to the study participants in the consent documentation and ethical approvals, and is compliant with relevant legal and regulatory requirements (eg, relating to data protection and privacy). The steering committee will have the right to review and comment on any draft manuscripts before publication. Data will be made available in line with the policy and procedures described at: https://www.ndph.ox.ac.uk/ data-access. Individuals wishing to request access should complete the form at https://www.ndph.ox.ac.uk/files/about/data_access_enquiry_form_13_6_2019.docx and email to: data.access@ndph.ox.ac.uk.

## Research in context

### Evidence before this study

Randomised trials have shown that nirmatrelvir–ritonavir and, to a lesser extent, molnupiravir reduce the risk of hospitalisation and death in individuals with early SARS-CoV-2 infection who are non-immune and at high risk of progression to severe disease, but evidence for efficacy in hospitalised patients is insufficient. We searched MEDLINE, Embase, and medRxiv between Sept 1, 2019, and April 26, 2024, for randomised controlled trials comparing the effect of molnupiravir or nirmatrelvir versus usual care or placebo on clinical outcomes in patients hospitalised with COVID-19. We used the search terms (Coronavirus infection OR COVID OR COVID-19 OR COVID19 OR 2019n-CoV OR SARS-COV-2 OR SARSCoV2 OR SARS-Cov2) AND (molnupiravir OR Lagevrio OR nirmatrelvir OR Paxlovid). No language restrictions were applied. We identified one trial that tested molnupiravir in 304 patients hospitalised with COVID-19, which we assessed as being at a low risk of bias (the MOVe-IN trial). This trial found no difference in the primary outcome of recovery by day 29 (82–85% in the molnupiravir groups *vs* 85% in the placebo group) or mortality (6% in the molnupiravir group *vs* 3% in the placebo group), but was underpowered to rule out clinically relevant improvements in either outcome. We also identified one trial testing nirmatrelvir–ritonavir in 264 hospitalised patients, which we assessed as being at a low risk of bias. In that trial there was no difference in the primary outcome of 28-day mortality (4% in the nirmatrelvir–ritonavir group *vs* 6% in the standard treatment group), but it was underpowered to rule out a clinically meaningful benefit of treatment. Details of the search and bias assessment are in the appendix (p 34).

### Added value of this study

We report the second ever randomised evaluations of both molnupiravir and nirmatrelvir–ritonavir in hospitalised patients from the Randomised Evaluation of COVID-19 Therapy (RECOVERY) trial, including three times more participants than previously reported for molnupiravir. During the period of recruitment, 770 (83%) of 927 participants were vaccinated against SARS-CoV-2, and almost all infections were with omicron variants. Despite evidence of increased viral clearance with both antivirals, we found no evidence of benefit of either antiviral on major clinical outcomes, although we cannot exclude a moderate benefit as this would require a larger trial. In the molnupiravir comparison, 74 (17%) of 445 participants allocated to molnupiravir and 79 (17%) of 478 participants allocated to usual care died within 28 days (hazard ratio [HR] 0·93 [95% CI 0·68–1·28], p=0·66). In the nirmatrelvir–ritonavir comparison, 13 (19%) of 68 participants allocated to nirmatrelvir–ritonavir and 13 (19%) of 69 participants allocated to usual care died within 28 days (HR 1·02 [0·47–2·23], p=0·96).

### Implications of all the available evidence

Current evidence does not support the addition of molnupiravir or nirmatrelvir–ritonavir to usual care, which could include other antivirals, for adults hospitalised with COVID-19. However, a clinically meaningful benefit of treatment cannot be excluded, particularly for nirmatrelvir–ritonavir.
